# Joint attention and its linguistic representation in dialogue: *embodiment* revisited

**DOI:** 10.3389/fpsyg.2023.1202455

**Published:** 2023-07-26

**Authors:** Guocai Zeng

**Affiliations:** College of Foreign Languages and Cultures, Sichuan University, Chengdu, China

**Keywords:** embodiment, joint attention, commitment, common ground, repetition

## Abstract

Lakoff and Johnson, among many others, have discussed the role of the human body in structuring meaning in communication, aiming to reveal the interrelation between the human body, language, and cognition. This study revisits the concept of *embodiment* and investigates its interactive nature functioning in speakers constructing repeated structures in conversation, based on the hypothesis made in this work that the joint attention of interlocutors essentially indicates the interaction of their embodied experience of the language used in the situated context, where speakers not only share their propositional commitments but also make individual contributions to establishing common ground in dialogue. Viewed in this way, at the linguistic level, the implicitly and/or explicitly repeated language resources displayed between utterances are in fact the encoding of speakers’ co-construction of joint attention and demonstrate the interplay of speakers’ syntactic and pragmatic knowledge in producing utterances in the talk turns. This research hopefully sheds some light on studies concerning the relationship between language and cognition as well as how language is constructed in dialogue from the interactive view of the syntax–pragmatics interface.

## 1. Structuring language and meaning: an *embodiment* view

In line with the theoretical view of the cognitive-functional approach to natural language, meaning constructed in language communication is fundamentally rooted in the speaker’s experience of the objective world,^1^ thus suggesting the epistemic stance that a speaker holds towards the entities s/he physically or mentally experiences in the reality. In this sense, language used to encode a speaker’s knowledge is embodied. For this study, the term *embodiment*, basically denoting that the human body matters when a speaker constructs his/her language, is construed mainly from two aspects, one of which is that in a broad sense a speaker understands the reality by dint of his/her bodily interaction with the objective world, while the other, in a more narrow sense and much more important, is that in daily conversations the *language* itself is the most crucial object speakers experience more frequently than other entities when they construct their dialogues. The latter point significantly indicates the embodied experience-based strategy that interlocutors employ when they take language to structure language in dialogue (*cf.* Du Bois, 2014; Zeng, 2021). The experience-based view of meaning construction, distinct from the rule-based structuring of meaning in the generative tradition of language studies, entails that meaning is personalized but coordinated between speakers in the communication. However, findings on how the human body functions in structuring language from a dialogic view are still rarely seen.

This study is supposed to bridge this gap to a certain degree by investigating the interactive nature of *embodiment* that virtually functions as the basis for interlocutors to establish joint attention, through which they produce their utterances in language communication. For this account, this research closely looks at the repeated structures in paired utterances that are produced by different speakers, proposing that explicitly and/or implicitly repeated grammatical structures are in essence the linguistic representation of the joint attention of interlocutors in dialogue, significantly indicating the speaker’s perception-based strategy of taking language to make language in dialogue.

## 2. Extant views on *embodiment*

According to Bergen (2015, p.11), in general, *embodiment* seems to be used to mean something about how the mind relates to the body. And in the view of Smith (2017, p.1), embodiment—having, being in, or being associated with a body—is a feature of the existence of many entities, perhaps even of all entities. For Rohrer (2007, p.27), in its broadest definition, the embodiment hypothesis is that human physical, cognitive, and social embodiment grounds our conceptual and linguistic system, while in the view of Walsh (2020), embodiment is a bi-directional link between the body and body language, where the body both demonstrates and creates our being. For cognitive linguists, language, as part of humans’ cognition, is fundamentally motivated by embodied experience (Wen and Jiang, 2021).

Remarkably, Rohrer (2007, p. 28–31) proposes that, with respect to human’s cognition, the term *embodiment* can be used in at least 12 different important senses, including Lakoff’s (1987, p. xiv) view that the core of our conceptual systems is directly grounded in perception, body movement, and experience of a physical and social nature. In line with Lakoff and Johnson’s (1999, p. 37) interpretation, the very properties of concepts are created as a result of the way the brain and body are structured and the way they function in interpersonal relations and in the physical world. Lakoff and Johnson (1999) also suggest that there are three levels of embodiment which together shape the embodied mind, namely the neural level, the level of phenomenological conscious experience, and that of the cognitive unconscious. In addition, Shapiro (2014), taking both empirical and philosophical views, investigates the properties of embodied cognition, especially focusing on such themes as embedded, extended, and enactive cognition, with the finding that there are strong interrelations between language and perception, reasoning, social and moral cognition, emotion, and consciousness, as well as human memory.

These findings have undoubtedly expanded our views about *embodiment* from different theoretical standpoints. But closer scrutiny of these studies suggests that most of the discussion on embodiment is not conducted from a dialogic view. This research will hopefully make a certain contribution to shortening this gap.

## 3. Interactive *embodiment*

Utterances in dialogue are interactive in nature. Thinking in this pattern, it is natural to describe and explain how language is produced from an interactive embodiment view. To be specific, language is constructed based on a speaker’s sharing of individually embodied experience of how language is used.

### 3.1. Language: the object speakers experience in dialogue

In the studies on the relationship between language and cognition, it is assumed that the human body plays a key role in language production and comprehension (e.g., Lakoff, 1987; Langacker, 1987, 1991; Lakoff and Johnson, 1999; Rohrer, 2007), with the most concern about a single speaker’s embodied experience of the physical world. But from the view that language is used dialogically (Bakhtin, 1981; Pickering and Garrod, 2021), how the experiences of speakers interact and what the role of such interaction could be in language production in conversation are in fact not paid much attention.

Unarguably, it is interlocutors^2^ who participate in the *embodiment* process to structure utterances used in communication. In this process, typically a speaker first perceives the object(s) based on his/her body interacting with the physical world, then narrows down his/her attention to certain aspect(s) of the given entity, which is also the grounding process of abstract conceptual content in the speaker’s mind (cf. Langacker, 2008). The consequence of this grounding is eventually mapped onto the grammatical structures of the utterances, showing the linguistic encoding of one’s experiencing of the reality.

Strikingly, also in this process, the language resources, which can be lexical items, sentence structures, functions, or prosodies of utterances used previously by a speaker, are the objects another speaker experiences physically or mentally. To put it another way, not only the human body but the language used to depict human’s embodied experiencing of the world is exactly the object humans interact with in daily conversations, and should be highlighted when the *embodiment* view of language is examined. Convincing evidence for this observation is the language phenomena of repetition, which fundamentally refer to a speaker’s imitation of his/her dialogic partner’s speech acts (Clark, 1977; Bybee, 2006; Huang, 2010) or a form of structural priming effect (Pickering and Garrod, 2021) in dialogue. Grounded on such imitations or structural priming, interlocutors verify their own comprehension of the reality in the interaction of embodied and individualized experience.

### 3.2. Interaction: the core of the concept *embodiment*

As Zlatev (2017) proclaims, the significance of examining embodied intersubjectivity can never be overestimated. Wen and Jiang (2021, p.150) hold a similar view that linguistic conceptualization is embodied in social interaction. To put it more simply, language is interactively embodied. The embodiment view of language is not only structured on the interaction of a single speaker’s body with the physical world, but also built on the interaction between interlocutors’ experiencing their dialogic partners’ language uses. To consolidate this view, how the joint attention of speakers in conversation is framed and then linguistically encoded is especially surveyed in the following sections.

### 3.3. Joint attention: how interactive *embodiment* works

Language communication is driven by the speaker’s attention (Langacker, 1984; Levelt, 1993; Giora, 2003; Myachykov and Posner, 2005; Talmy, 2007, 2017; Breyer, 2009; Shtyrov et al., 2010; Lampert, 2015; Yliniemi, 2021; Dash et al., 2022). This view might work to instantiate the principle of ‘*what you see is what you get* (e.g., Boas, 2021, p.64)’ followed in cognitive linguistic studies on natural language. With this thinking in mind, dialogic partners’ experiencing of each other’s language use is in fact the basis for interlocutors to structure joint attention in conversation.

#### 3.3.1. Attention in dialogue

Attention is the window through which a speaker perceives the world based on his/her body. A speaker’s attention in dialogue might be visual attention or mental attention. Both of them are structured on human organs, particularly the former on eyes and the latter on the mind. The scope a speaker’s attention can reach is roughly classified into the maximal scope, immediate scope, and the focused area (Langacker, 2008, p. 260–263).

When it comes to a speaker’s talking about something verbally or non-verbally, s/he directs her or his own visual or mental attention to the candidate instance(s) within a category of entities first, and then zooms the attention in to particular one(s), focusing on the feature(s) of the targeted instance. To attract the speaker’s attention, the candidate instance in a category is prototypically salient to a certain degree in the speaker’s physical or mental world. As Talmy (2021) argues in his analyses of attention phenomena, entities with more salience in terms of their locations and/or shapes, etc., are foregrounded while those which are less salient will be backgrounded in the conversational settings; those closely related to or participating in the ongoing dialogue process are more likely to be the center of the speakers’ attention; moreover, entities with a higher degree of complexity in the internal organization demand more cognitive processing efforts from the speaker, thus they are more likely to be attended to by a speaker; what is more, moveable objects rather than static ones in the attended scope are supposed to attract more attention from the interlocutors. Put briefly, entities prominent in certain features in the speaker’s attention scope are more likely to be qualified as the dialogic focus in communication.

#### 3.3.2. Joint attention

Zlatev (2017) proposes that meaning is sourced from humans’ interaction and is especially associated with speakers’ interactive and enactive perception, when he expounds embodied intersubjectivity in general and mimetic acts in particular, based on the analyses of body schema, body language, body memory, bodily movement, and perception. This study goes further, assuming that interactive perception is the speaker’s bodily ground to co-construct joint attention in dialogue.

**Figure 1:**
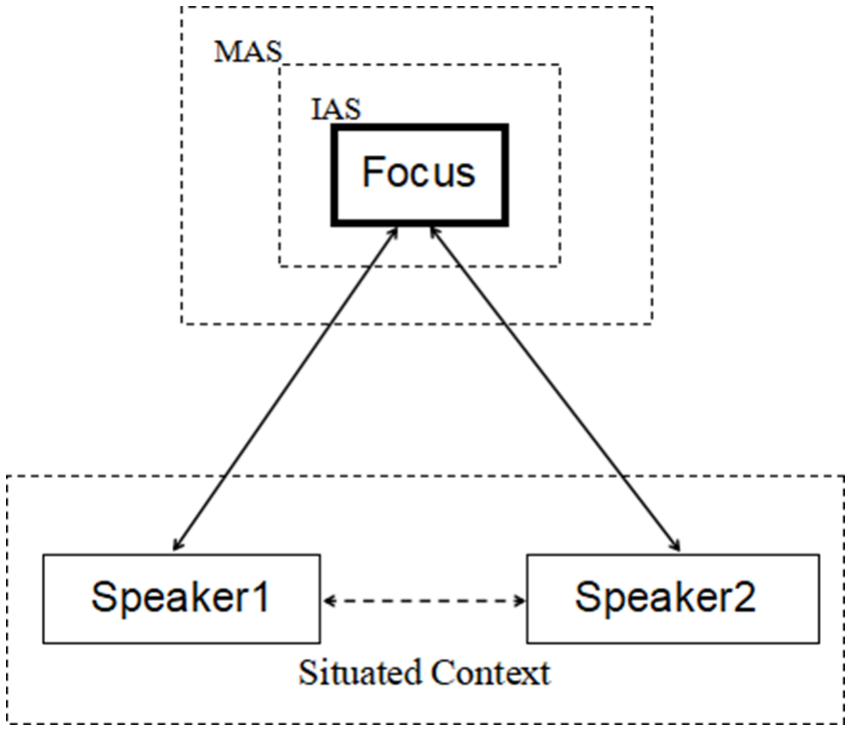
The frame of joint attention in dialogue.

Joint attention, by definition, is the mental window shared by speakers (*cf.* Tomasello, 1995; Clark and Josie, 2008; Kecskes, 2008; Mondada, 2009) and it is interactively embodied in nature. To construct the joint attention, the speaker grounds the salient entity in his/her visual or mental world, and then directs the hearer’s attention to the same entity via language or non-language cues. In doing so, an overlapping of attention from both the speaker and the hearer is displayed in the conversation. Before joint attention is formed, interlocutors do have their own focuses of attention. For this reason, what is attentively aimed at in joint attention might differ from the one that is the individual speaker’s concern. The process where an object is attentively targeted by both speaker and hearer reveals the interlocutors’ cognitive coordination to establish the ground for ongoing dialogic interactions. The frame of joint attention can be illustrated as in Figure 1.^3^

Figure 1 shows that in conversational settings two speakers^4^ interact to shift their attentions to the same entity, namely the dialogic focus marked by the rectangle with solid bold black lines. This jointly attended entity is the one located in the immediate attention scope (short for IAS), where other entities related to the focused one but with less salient features in shape, location, or color are backgrounded in the maximal attention scope (short for MAS), which is the largest size of joint attention area. The MAS and IAS suggest the speakers’ different allocations of their attentions. That is, a speaker devotes least attention to the MAS and most to the focused entity. Rooted in the interactive perception-based joint attention scope, speakers make and share their propositional commitments towards the dialogic focus and progressively make contributions to enlarging the common ground, based on which the interlocutors coordinate their stance-takings in the taking of turns.

### 3.4. Beyond joint attention

In the process of building joint attention, a speaker makes propositional commitments about the targeted entity to his/her dialogic partner on the one hand (Katriel and Dascal, 1989; De Brabanter and Dendale, 2008; Gilbert, 2013; Heinonen, 2015; Bonalumi et al., 2020; Elder, 2021), and contributes to constructing the common ground shared by the interlocutors in conversation on the other hand.

#### 3.4.1. Propositional commitment shared

According to Geurts (2019, p.1), human communication is first and foremost a matter of negotiating commitments and every speech act causes the speaker to become committed to the hearer to act on a propositional content. On his account, commitment is a three-place relation between two individuals, *a* and *b*, and a propositional content, *p.* That is, *a* is committed to *b* to act on *p* (*ibid*:3). Commitment is therefore understood as a social relationship subserving action coordination between individuals. Following this view, in the zone of joint attention, the speaker’s language coding of events *de facto* makes an epistemic commitment (*cf.* Hoff, 2019) concerning specific propositional contents to the hearer, who in turn makes a propositional commitment towards the speaker by producing a responsive utterance (*cf.* Boulat, 2014).

From a dialogic view, the interlocutor’s mutual commitments are joint attention-based in that the proposition contents in commitments are related to entities that are mentally contacted by both speaker and hearer, whereas a single speaker’s propositional commitment is not insofar as there is no engagement of speakers’ interaction. In the joint attention zone, a speaker might have strong or weak commitment toward the shared target, suggesting the different allocations of attention of the interlocutors in conversation. The speaker’s epistemic stance towards the dialogic focus could be weakened, reaffirmed, or even completely undermined because of the dialogic partner’s propositional commitment to it, denoting the degrees of speakers’ subjectivity in construing the object in the attention scope and reflecting the different consequences of the speakers’ experiencing of the reality.

Therefore, in the exchange of talk turns, speakers actually share their propositional commitments to the jointly attended objects. The commitment interaction, which is speaker-centered (Moeschler, 2013) or hearer-based (Morency et al., 2008), is then justified as the engine driving the dialogue process to go forward. Since joint attention is structured in humans’ perception interaction, the shared commitment made in the dialogue is substantially embodied.

#### 3.4.2. Common ground established

In the joint attention, the commitment made by a speaker suggests his/her knowledge about the world. Such knowledge contributes to the structuring of the common ground^5^ in developing the size of local dialogue (Stalnaker, 2002; Abbott, 2008; Kecskes and Zhang, 2009, 2013; Allan, 2013; Green, 2017; Semeijn, 2017; Swanson, 2020; Marsili, 2021). Particularly, according to Kecskes and Zhang (2009, p.347), there are two sides to common ground: *core common ground* and *emergent common ground*. In their view, the former refers to the relatively static, generalized, shared knowledge that belongs to a particular speech community, while the latter designates the relatively dynamic, specific, private knowledge created in the progress of communication that belongs to the individual(s).

In a broad sense, common ground encapsulates conventional social-cultural information that is by default understood by interlocutors and their personally embodied experience in the world (Jaszczolt, 2005, 2016). Narrowly speaking, what is emergent in the ongoing speakers’ interaction could be the newly built common ground knowledge, some of which might be much more salient in the focused and/or the immediate attention area than that in the maximal attention scope.

Kecskes and Zhang (2009) also propose that the individual attention, which is the cause of the interlocutor’s egocentrism, and the speaker’s intention, which through relevance is expressed in cooperation, are equally important in constructing common ground. From an interactive embodiment view of the speaker’s attention, common ground is naturally constructed through the process where speakers with egocentric behaviors interact with each other to build interpersonal cooperation. The more commonalities speakers construct with collaborative efforts in the joint attention zone, the more opportunities interlocutors have for achieving agreement in the negotiation of stances.

## 4. Repetition^6^: the linguistic representation of joint attention

Rohrer (2007, p. 26) mentions *using language to establish joint attention*. Tomasello and Farrar (1986), Krause (1997), Charman (2003), Robins et al. (2004), Eilan et al. (2005), Diessel (2006), and Skarabela (2007), among others, have also investigated the relationship between language and speaker’s attention, but how grammatical structures linguistically encode joint attention in conversation has not been examined with great detail.

As Figure 1 implies, language emerges in dynamic conversation, in which the interaction of the speakers’ embodied experience occurs. Linguistically speaking, grammatical structures that are shared by speakers in such interaction basically encode the mutual commitments made and the common ground shared by speakers. Or more precisely, repetitions of language resource in dialogue function to highlight the embodied interaction of individual attention, displaying how joint attention is co-constructed by different speakers in talk turns, which can be elaborated in dialogue (1).

Dialogue (1)^7^

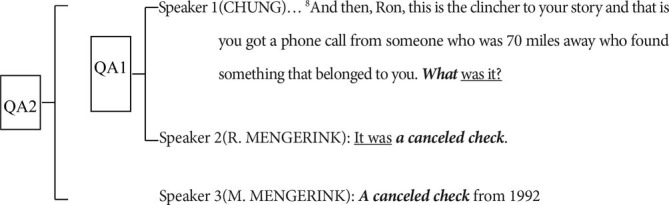

  Diagraph for dialogue (1)^9^

**Table 1:** 

Speaker 1	↓	...	What	was	it	?
Speaker 2					It	
				was		
			a canceled check			.
Speaker 3			A canceled check from 1992			.

We can see from the diagraph for this local dialogue that there are three talk turns. At the end of Speaker 1’s talk turn is a wh-question ‘*what was it?*’. Cognitively speaking, in contrast with the previous utterance that works as the background for speaker 1 to produce this wh-question, the *what* is more saliently positioned in the conversation because of its unspecified semantic content in this question. The utterance backgrounded for the wh-question in the first talk turn is in the maximal attention scope of speakers, and works as the common ground for speakers 2 and 3 to construe the instantiation of *what,* which is in the immediate attention scope of all speakers.

From an interactive view of embodiment, the heading position and the schematic content of *what* in the question are the motivation for speakers 1–3 to construct joint attention, whose focus is exactly the *what*. In speaker 2’s talk turn, *what* is specified as *a canceled check,* while it is *a canceled check from 1992* in speaker 3’s talk turn, a slightly more detailed instance of *what*, demonstrating the process in which speakers 2 and 3 make their own propositional commitments to the schematic *what*. By doing so, the semantic content of *what*, the jointly attended target by the interlocutors in the ongoing conversation, is specified step by step with finer details.

Furthermore, the explicitly paralleled structures *‘it:it; was:was’* within the question-answer 1(QA1) and ‘*what*: *a canceled check’* in QA 2*,* as well as the symmetrical structures ‘*a canceled check: a canceled check’* between speakers 2 and 3’s talk turns, work together to indicate the common grounds for the speakers to further interpret *what.* The syntactic parallelism in this sense significantly reveals the linguistic evidence of the speakers’ joint attention on *what*. The alignment of speakers’ attention at the same time signifies that three speakers successfully build cooperation to specify the schematic content of *what* as *a canceled check* and *a canceled check from 1992*.

The way that joint attention is constructed in dialogue is also observed in child-to-child interaction, as shown in dialogue (2), a case of Mandarin-speaking children’s conversation.

Dialogue (2): (Loc:Chinese/Mandarin/LiZhou/3/06.cha)^10^
  @ID: zho|LiZhou|CH1|3;00.|female|||Target_Child|||
  @ID: zho|LiZhou|CH2|3;00.|male|||Target_Child|||
  342 CH1^11^: 你 这 个 会 唱 歌 吧?
  Ni zhe ge hui chang ge ba?
  You this can sing song PARTICLE.
  ‘Can your this one sing songs?’
  343 CH2: 这 不 会 唱 歌。
  Zhe bu hui chang ge.
  This cannot sing song.
  ‘This one cannot sing songs.’
  344 CH1: 这 个 会 唱 歌。
  Zhe ge hui chang ge.
  This one can sing song.
  ‘This one can sing songs.’
  345 CH2: 老虎 的 歌。
  Laohu de ge.
  Tiger PARTICLE song
  ‘Songs about tigers.’
  346 CH1: 老虎 的 歌。
  Laohu de ge.
  Tiger PARTICLE song
  ‘Songs about tigers.’
  347 CH2: 哈哈。
  Ha ha.
  PARTICLE.
  ‘Ha-ha.’
  Diagraph for dialogue (2)

**Table 2:** 

			particle	personal pronoun	demonstrative pronoun	negation	modal word	verb	noun	auxiliary word	noun	article	
342	CH1:	↓		Ni	*zhe ge*		*hui*	*chang*			*ge*	ba	?
343	CH2:				*zhe ge*	bu	*hui*	*chang*			*ge*		.
344	CH1:				*zhe ge*		*hui*	*chang*			*ge*		.
345	CH2:								*Laohu*	*de*	*ge*		.
346	CH1:								*Laohu*	*de*	*ge*		.
347	CH2:		Haha										.

As demonstrated in this diagraph, repetitions of words (marked as italics) are obviously produced along with the ongoing dialogue process, simultaneously displaying the emergent shared template structures ‘*pronoun + modal word + verb + noun*’ and ‘*noun + auxiliary word + noun*’, with ‘*zhe ge hui chang ge (this one can sing songs)*’ and ‘*laohu de ge (songs about tigers)’* as the instances. These patterned structures, jointly attended to by these two child speakers, are also the common grounds for them to develop the size of the local discourse.

To be more specific, within talk turns 342–344, both of them focus on the schematic event ‘*X hui Y (X can do Y)*’ and its instance ‘*zhe ge hui chang ge (this one can sing songs)*’, while in talk turns 345–346, child 1 and child 2 attend the same and more specific object, which is ‘*laohu de ge (songs about tigers)*,’ revealing the cognitive coordination and interpersonal cooperation between the child interlocutors by making commitment to each other. In the last talk turn (347), the particle ‘*haha*’ indicates that, at the end of this episode of a short conversation, child 2’s attention is successfully directed by child 1 to the instance of ‘*ge (songs)*’, namely *laohu de ge (songs about tigers)*.

In this sense, repeated structures are viewed as the linguistic encoding of the joint attention of speakers in dialogues (1) and (2), which at the meantime implies the interplay of interlocutors’ syntactic-pragmatic knowledge in structuring utterances, thus bringing forth dialogic resonance (*cf.* Du Bois, 2014) in communication, as can be further observed in dialogue (3).

Dialogue (3) (*Tastes Very Special* SBC031: 533.430–541.201)^12^
  1 SHERRY; @^I @don’t even like **ice** ^**tea**.
  2 BETH; (H) (0.7) Do you like ¿^**hot tea**?
  3 (0.6)
  4 SHERRY; ^Yeah,
  5 I ^love **hot tea**.
  Diagraph for dialogue (3)

**Table 3:** 

			pronoun	auxiliary verb	verb	noun	punctuation
SHERRY	↓		*I*	*do not*	*like*	*ice tea*	.
BETH				*do*			
			*you*		*like*	*hot tea*	?
SHERRY		Yeah					,
			*I*		*love*	*hot tea*	.

As seen in this diagraph, SHERRY first made a commitment with a negative statement introducing ‘*ice tea*’. SHERRY’s negative attitude towards *ice tea* primarily functions as the partial common ground for BETH to structure her expression containing ‘*hot tea*’. The partially repeated structures ‘*ice tea: hot tea*’ based on ‘*tea*’ shows that both speakers have mental contact with ‘*tea*’. That is to say, the categorical entity encoded by *tea* is jointly attended by the two speakers when they make speech acts concerning SHERRY’S preference of the instance of *tea*. Meanwhile, this parallelism suggests SHERRY and BETH have their own allocation of attention in the joint attention scope, with the former on *hot tea* but the latter on *ice tea*.

Notably, the shared structural pattern ‘*X like Y*’ abstracted in talk turns 1–2 based on the joint attended ‘*tea*’ is the background for SHERRY to structure her utterance ‘*I love hot tea.*’ The partial symmetry in the semantic structure between *love* and *like,* which in this case indicates a certain degree of preference for *tea*, also signifies the interpersonal interaction founded through the structural alignment ‘*I: you: I.*’ In addition to that, grounded on this grammatical pattern, the two speakers’ embodied interactive stance-takings are entirely presented via the negative tone of talk turn 1, the interrogative tone in talk turn 2, and the assertion in the last talk turn, altogether displaying the pragmatic function of joint attention in the dialogue.

## 5. Concluding remarks

To sum up, as Figure 1 and dialogues (1)–(3) suggest, utterance interaction in conversation entails the speakers’ co-construction of joint attention, which is rooted in the speaker’s general ability to perceive the world through the human body. Language production is hence driven by the formation of the speaker’s joint attention, that is, the interaction of individual attention in the communication. More precisely, the interlocutors in dialogue take language to structure language through setting up joint attention. In doing so, the speakers at the same time make commitment to each other and establish common ground for the ongoing dialogue, based on their embodied experience of their partner’s language use in situated context. At the linguistic level, repeated language structures are in essence the encoding of speakers’ joint attention. In this sense, language is interactively embodied in nature. Human’s interactively embodied experience of other persons’ language use essentially reveals interlocutors’ cognitive coordination and interpersonal cooperation in communication. According to Wang (2019), the *embodiment* view of natural language, one of the fundamental claims in cognitive linguistic studies, cannot be overemphasized and the theoretical assumptions in *Cognitive Linguistics* should be reinterpreted or redefined within the framework of *Embodied Cognitive Linguistics*. This study will, hopefully, widen the views of the sense of *embodiment* from a dialogic perspective on language and shed some light on the research concerning the relationship between language and cognition as well as how language is constructed in dialogue from the interactive view of the syntax–pragmatics interface.

## Data availability statement

The original contributions presented in the study are included in the article/supplementary material, further inquiries can be directed to the corresponding author.

## Ethics statement

Ethical review and approval was not required for the study on human participants in accordance with the local legislation and institutional requirements. Written informed consent from the patients/ participants or patients/participants' legal guardian/next of kin was not required to participate in this study in accordance with the national legislation and the institutional requirements.

## Author contributions

The author confirms being the sole contributor of this work and has approved it for publication.

## Funding

This work was supported by National Social Science Fund of China (Grant number: 18BYY076).

## Conflict of interest

The author declares that the research was conducted in the absence of any commercial or financial relationships that could be construed as a potential conflict of interest.

## Publisher’s note

All claims expressed in this article are solely those of the authors and do not necessarily represent those of their affiliated organizations, or those of the publisher, the editors and the reviewers. Any product that may be evaluated in this article, or claim that may be made by its manufacturer, is not guaranteed or endorsed by the publisher.

## Footnotes

^1^The ‘objective world (*cf.* Lakoff and Johnson, 1999)’ in this work is applied particularly in the sense of the physical world where human beings live.

^2^This study concerns typical conversations where the speaker and the hearer are different persons. A monologue, where the speaker and hearer is the same person, can be analyzed in the same way.

^3^This figure is based on the modifications of Figures present in Langacker (2008, p. 260-263).

^4^Only two speakers are mentioned here and they are two different persons. A trialogue or monologue can be analyzed in the same way.

^5^Common ground here is defined to include the emergent knowledge in the dialogue, which goes beyond the discussions by Stalnaker (2002) and Abbott (2008).

^6^Since the joint attention is constructed with different speakers’ efforts, other-repetitions rather than self-repetitions (*cf.* Huang, 2010) in dialogue are concerned in this study.

^7^This dialogue is retrieved from COCA corpus (source-SPOK:CNN-Chung; Date 2002-11-12; Title: Latest bin Laden Tape Stirs Debate, Fear of New Attacks).

^9^A diagraph (*cf.* Du Bois, 2014) is used here to indicate the structural mapping and parallelism between utterances and the symbol ‘↓’ in the diagraph indicates the direction of turn-construction.

^10^This dialogue is retrieved from CHILDES corpus (MacWhinney, 2000).

^11^CH1 and CH2 here refer to child-speaker 1 and child-speaker 2, respectively.

^12^This dialogue is quoted from Du Bois (2014, p. 381).
